# Vitamin - D supplements for better osteointegration in dental implant surgery: A randomized control trial

**DOI:** 10.6026/973206300211245

**Published:** 2025-05-31

**Authors:** Abhishek Kumar Singh, Hiralal Ash, Ajay Kumar Singh, Sujay Kumar Sinha, Rajeev Kumar Singh, Kumar Arunesh

**Affiliations:** 1 Department of Oral and Maxillofacial Surgery, Budhha Institute of Dental Sciences and Hospital, Patna, Bihar, India

**Keywords:** Implant, osseointegration, Vitamin - D

## Abstract

The role of Vitamin - D in osseointegration is of interest. Henceforth, the randomized control trial was conducted wherein 40 Participants were equally divided into two groups. Group A received Vitamin - D3 for 12 weeks following implant placement while Group B, did not receive Vitamin - D supplementation. Osseo-integration was evaluated using radiographic and clinical assessments with Ostell (ISQ). Significant association between Vitamin - D levels and the osseointegration of dental implants was observed. Thus, the relationship between Vitamin - D and dental implant success is shown.

## Background:

Prosthetic rehabilitation of missing structures in the oral and maxillofacial region in accordance with DeVan's principle of preservation has been the ultimate challenge to the prosthodontics. Over the years, traditional methods of tooth replacement are slowly and steadily being replaced by newer modalities like implants [1]. Implants provide for rehabilitation and restore function and esthetic needs in patients with missing teeth. It is essential to have sufficient bone bulk and density at the implant site in order to achieve good bone - to- implant contact and primary stability, which are crucial for osseointegration [2]. Many factors play a significant role in osseointegration and healing after dental implant insertion and restoration. Some factors are related to dental biomaterials, such as the dental implant, prosthesis and grafting materials. Other factors can be connected to operator skills and accumulated experience. Local and systemic patient-related factors are crucial in determining the success of the dental implant. The patient's systemic condition directly affects the healing of the dental implant. One of the most overlooked systemic factors influencing bone formation around the implant and subsequent osseointegration around the implant includes Vitamin - D level [3]. Vitamin - D, a fat-soluble vitamin that plays a vital role in regulating calcium and phosphate metabolism. It makes it a key player in bone remodeling and repair. It promotes the differentiation and activity of osteoblasts, cells responsible for bone formation and modulates osteoclasts, which resorb bone tissue. Therefore, adequate levels of Vitamin - D are not only essential for maintaining bone density, but also for enhancing the healing process in bone tissues affected by dental implant procedures [4]. Vitamin D supplements may support better implant integration in patients with low Vitamin D levels, but more research is needed to confirm their overall effectiveness in dental implant success [5, 6]. Therefore, it is of interest to evaluate the role of Vitamin - D in osseointegration.

## Materials and Methods:

The randomized control trial included 40 patients aged 21 to 55 years, either gender with Vitamin - D insufficiency undergoing dental implant procedures. Exclusion criteria was prior bone regenerative therapy, arthritis, periodontal disease, bleeding disorders, poor oral hygiene, uncontrolled hypertension and diabetes mellitus, severe Vitamin - D deficiency, chronic renal disease, use of proton pump inhibitors, pregnant women. 40 Participants were equally divided into two groups: Group A consisted of 20 patients who received a daily oral intake of 5000 IU Vitamin - D3 for 12 weeks following implant placement while Group B, control consisted of 20 patients who did not receive Vitamin - D supplementation following implant placement. Osseo-integration was evaluated at three months using radiographic and clinical assessments with Ostell (ISQ). Additionally, serum Vitamin - D levels were measured pre-operatively and post-operatively at 3 months interval post implant placement. Data analysis was performed using SPSS version 23.00.

## Results:

The mean age of the study sample was 43.37±7.44 years. Results are described in Table 1, which shows the distribution of study participants based on the status of bone after 3 months of follow-up among two groups. The median ISQ at three months was notably higher in Group A (Vitamin - D supplement) compared to Group B (placebo), with values of 75 vs. 62 ISQ (p=0.001). Intergroup comparison of bone in Group A and B before and after follow up of 3 months Moreover, the median Vitamin - D levels at three months were significantly elevated in Group A compared to Group B (p=0.001). The study found that Vitamin - D levels were significantly higher in Group A compared with Group B at three months (p=0.001) that suggesting that the supplementation was effective in elevating Vitamin - D levels. Hence, the present study, revealed significant association between Vitamin - D levels and the osseointegration of dental implants.

## Discussion:

Epidemiological data suggest that Vitamin - D deficiency is widespread, affecting about 1 billion people worldwide. This deficiency is especially prevalent in older adults, a group that also forms a significant portion of the dental implant recipient population [5, 6-7]. Current evidences suggest that while Vitamin - D status may have a role in bone health and its association with several risk factors can impact the osseointegration failure. Hence, its direct impact on dental implant osseointegration remains inconclusive. Henceforth, present study was conducted to evaluate the role of Vitamin - D in osseointegration. Results revealed significant association between Vitamin - D levels and the osseointegration of dental implants. Vitamin - D is a secosteriod hormone essential for calcium absorption and bone mineralization which is positively associated with bone mineral density [8]. The role of Vitamin - D in producing more anti-inflammatory cytokines and less pro-inflammatory cytokines is crucial during the first few weeks after implant surgery as this reduces the body response to surgery. During osteointegration of the implant, a satisfactory concentration of Vitamin - D is preferable because of the intense processes of resorption and osteogenesis [8]. Studies suggest low Vitamin - D levels may negatively impact the process of osseointegration after implant placement, but studies have also reported the lack of a definitive link between low serums Vitamin - D levels and implant failure. Therefore, the association remains controversial. Some studies suggest that 1, 25-dihydroxyVitamin - D3 positively affects cell differentiation and matrix mineralization and thus, may act as a stimulating factor in osteoblastic bone formation [9, 10-11]. Kwiatek *et al.* in their high quality randomized trial noted a significant increase in some density following Vitamin - D supplementation [12]. Werny, 2022 revealed that Vitamin - D deficiency had a negative effect on the osseointegration of implants in animals. The supplementation of Vitamin - D appeared to improve the osseointegration in animals with systemic diseases, such as Vitamin - D deficiency, diabetes mellitus, osteoporosis and chronic kidney disease *etc.* [6] which was similar to that reported in present study. Pereira *et al.* shows 9.8% implant loss rate and its impact might indicate a more complex interaction between Vitamin - D and bone remodeling [13]. This was not in accordance to that reported in present study. Hence, further prospective clinical research studies as well as randomized controlled trials are needed to show more validation and correlation between decreased serum levels of Vitamin - D and increased risk of dental implant failure in perspective of Vitamin - D supplementation which can promote the osseointegration of dental implants.

## Conclusion:

The role of Vitamin - D and its direct impact on dental implant osseointegration remains inconclusive. We show a revealed significant association between Vitamin - D levels and the osseointegration of dental implants. Further prospective clinical research studies as well as randomized controlled trials are needed to show more validation and correlation between decreased serum levels of Vitamin - D and increased risk of dental implant failure in perspective of Vitamin - D supplementation which can promote the osseointegration of dental implants.

## Figures and Tables

**Table 1:** Frequency distribution of study participants based on status of bone after 3 months follow-up among two groups

**Groups**	**Bone**								**Total**		**P value**
	**No change**		**1-2 mm bone loss**		**2-3 mm bone loss**		**Bone regeneration**				
	**n**	**%**	**n**	**%**	**n**	**%**	**n**	**%**	**n**	**%**	
Group A	3	15	5	25	0	0	12	60	20	100	
Group B	0	0	3	15	17	85	0	0	20	100	0. 001
